# Apomixis and Hybridization Drives Reticulate Evolution and Phyletic Differentiation in *Sorbus* L.: Implications for Conservation

**DOI:** 10.3389/fpls.2018.01796

**Published:** 2018-12-13

**Authors:** Tracey J. Hamston, Natasha de Vere, R. Andrew King, Jaume Pellicer, Michael F. Fay, James E. Cresswell, Jamie R. Stevens

**Affiliations:** ^1^Molecular Ecology and Evolution Group, Biosciences, College of Life and Environmental Sciences, University of Exeter, Exeter, United Kingdom; ^2^Field Conservation and Research Department, Whitley Wildlife Conservation Trust, Paignton, United Kingdom; ^3^National Botanic Garden of Wales, Llanarthney, United Kingdom; ^4^Faculty of Earth and Life Sciences, Aberystwyth University, Aberystwyth, United Kingdom; ^5^Jodrell Laboratory, Royal Botanic Gardens, Kew, United Kingdom; ^6^School of Plant Biology, University of Western Australia, Crawley, WA, Australia

**Keywords:** apomixis, conservation, diversification, evolution, hybridization, polyploidy, *Sorbus*

## Abstract

Hybridization and polyploidy are major forces in the evolution of plant diversity and the study of these processes is of particular interest to understand how novel taxa are formed and how they maintain genetic integrity. *Sorbus* is an example of a genus where active diversification and speciation are ongoing and, as such, represents an ideal model to investigate the roles of hybridization, polyploidy and apomixis in a reticulate evolutionary process. To elucidate breeding systems and evolutionary origins of a complex of closely related *Sorbus* taxa, we assessed genotypic diversity and population structure within and among taxa, combining data from nuclear DNA microsatellite markers and flow cytometry. Clonal analysis and low genotypic diversity within the polyploid taxa suggest apomixis is obligate. However, genetic variation has led to groups of ‘clone-mates’ within apomictic taxa that strongly suggest mutation is responsible for the genotypic diversity of these apomictic lineages. In addition, microsatellite profiles and site demographics suggest hybridization events among apomictic polyploid *Sorbus* may have contributed to the extant diversity of recognized taxa in this region. This research demonstrates that both macro- and micro-evolutionary processes are active within this reticulate *Sorbus* complex. Conservation measures should be aimed at maintaining this process and should therefore be prioritized for those areas of *Sorbus* species richness where the potential for interspecific gene flow is greatest.

## Introduction

Hybridization between species resulting in the formation of new polyploid populations that are distinct and reproductively isolated from the parental taxa is the most common mechanism for sympatric speciation (Grant, 1981; Mallet, 2007). However, the frequency and the main formation routes of polyploid taxa remain unclear (Soltis et al., 2010) and studies of hybridization processes in polyploid species complexes may help to understand this form of speciation.

Apomixis (synonymous with agamospermy; asexual seed production) is often associated with polyploidy (Whitton et al., 2008) and effectively causes instant reproductive isolation of novel polyploids from sexual progenitors, enabling sympatric establishment while maintaining the heterozygosity associated with hybridization. Where apomixis is partial or facultative it allows for occasional exchange of genetic material where such apomicts co-occur with sexual counterparts (Richards, 2003). Apomictic groups develop an intricate variety of morphologically uniform clonal lineages which may be designated as species or microspecies (Grant, 1981), hence leading to much debate over species delineation; examples include: *Rubus* L. (Newton, 1980), *Taraxacum* (Hughes and Richards, 1988), *Crataegus* L. (Dickinson et al., 2008), and *Sorbus* L. (Rich et al., 2010). Hybridization, polyploidy and apomixis are all features of these and other complex genera and those groups that contain evolutionary young species represent good models to investigate the roles of these processes in plant speciation.

*Sorbus* (Rosaceae) is a suitable study group to test the extent of hybridization among species of varying ploidy and elucidate the role of breeding system in creation of novel polyploids and establishment of polyploid populations as the ongoing speciation in *Sorbus* is well described, particularly in Britain (Rich et al., 2010; Robertson et al., 2010). Sexual diploid taxa are thought to be pivotal in the creation of novel polyploids (Robertson et al., 2004, 2010; Hajrudinović et al., 2015b). Contact zones between sexual diploids and partially apomictic polyploids have produced a reticulation of allopolyploids (polyploids produced from interspecific hybridization) with varying fertility and ploidy levels (Ludwig et al., 2013; Hajrudinović et al., 2015a), In *Sorbus*, where polyploids are geographically isolated from diploids, the role of hybridization among allotetraploids and divergent mutation of polyploids, both of which may have contributed to the genetic diversity of the *Sorbus* complex has not been fully investigated.

The interest in this genus stems from its evolutionary biology and the conservation status of many *Sorbus* species. Several of the apomictic polyploid taxa are narrow-range endemics existing only in small populations; 12 United Kingdom species are threatened according to the International Union for Conservation of Nature [IUCN] (2016), making them a priority for conservation. Since the production of these endemic taxa relies on hybridization there is a growing awareness that process-based conservation is most appropriate, focusing on the evolutionary mechanisms that generate taxonomic complexity rather than a collection of possibly ill-defined individual taxa. Indeed, such a plan has been proposed for the endemic *Sorbus* of Arran, Scotland (Ennos et al., 2012). However, the development of appropriate conservation strategies depends on detailed knowledge of the processes concerned.

Several important United Kingdom sites for *Sorbus* diversity occur in the county of Devon and along the north Somerset coast (Rich et al., 2010). This study focuses on a group of seven polyploid taxa, four of which are narrowly endemic and of conservation concern. Their sexual diploid progenitors, *S. torminalis* (L.) Crantz and *S. aria* L., exist at low densities or are not currently native to the region, respectively.

**FIGURE 1:**
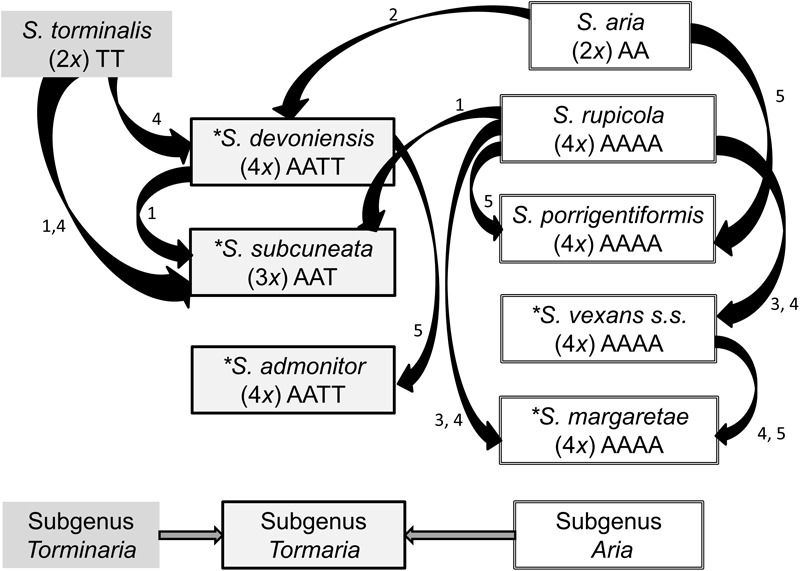
Previously hypothesized relationships among the study *Sorbus* species based on the following literature. 1. Wilmott (1934), 2. Sell (1989), 3. Proctor et al. (1989), 4. Chester et al. (2007), 5. Rich et al. (2010). Haploid genomes from *S. aria* and *S. torminalis* are indicated by the letters A and T, respectively (Richards, 1975). The summarized relationships among the three subgenera are also shown; *Sorbus torminalis* is the ancestral maternal parent for subgenus *Tormaria* and polyploid members of subgenus *Aria* are derived from *S. aria* (Chester et al., 2007). Assumed ploidy is in parenthesis, from Pellicer et al. (2012). ^∗^ Taxa largely restricted to study region.

*Sorbus rupicola* (Syme) Hedlund and *S. porrigentiformis* E.F. Warb. are thought to be the oldest polyploids in our study group based on their wide distribution (Rich et al., 2010), and are possible progenitors for other polyploid *Sorbus* in this region, as they have been shown to be in other areas (Robertson et al., 2004, 2010). *Sorbus vexans* E.F. Warb., *S. margaretae* M.C.F. Proctor, *S. admonitor* M.C.F. Proctor and *S. subcuneata* Wilmott are restricted to areas along the north coast of Devon and Somerset. *Sorbus devoniensis* E.F. Warb. is largely found in Devon, however, a number of individuals are found on sites in southeast Ireland (Rich et al., 2010). Previously proposed relationships among the study species are presented in Figure 1. Evidence for these relationships comes from both morphological (Warburg, 1962; Sell, 1989) and molecular studies; Nelson-Jones et al. (2002) used restriction fragment length polymorphisms (RFLPs) to assign hybridogenous polyploid *Sorbus* taxa to various subgenera, and plastid DNA identified the ancestral maternal parent (Chester et al., 2007). These taxonomic groupings accord with those defined on the basis of peroxidase isoenzyme studies (Proctor et al., 1989) which also suggested variation within some polyploid taxa. However, the hybrid origins of our study taxa, and in particular the pollen donors, were not identified.

**FIGURE 2:**
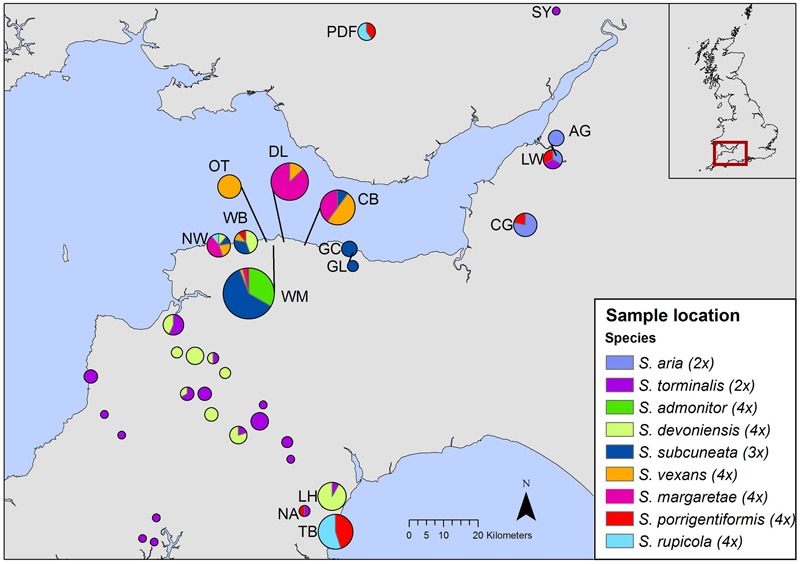
Geographic distribution of samples included in our study. Each pie chart represents a site, with pie size relative to site sample size. The inset shows the area covered by the map. Site codes match those in Supplementary Table S1 (The map was created using ArcGIS Desktop version 10.2.2, ESRI, Redlands, CA, United States, URL: http://www.esri.com/).

To differentiate among closely allied species with possible common ancestry, we employed nuclear DNA microsatellite markers; they are codominant and highly polymorphic (due to relatively high mutation rates), which makes them well suited for the identification of hybrid parentage (Freeland et al., 2011). We used flow cytometry to determine relative nuclear DNA contents and to infer ploidy for our species. Flow cytometry is a useful tool for rapid screening of samples and has been used increasingly to explore hybrid speciation (Pellicer et al., 2012; Hajrudinović et al., 2015b). Our sampling strategy sought to encompass the geographical ranges of *S. admonitor, S. subcuneata, S. devoniensis, S. vexans* and *S. margaretae*, whilst other potential parental species were sampled more widely to obtain a representative sampling of alleles for these taxa (Figure 2).

The principal aims of this study were to elucidate evolutionary relationships among the study taxa and to determine breeding systems within this species complex; in addressing these aims, we explored patterns of genetic structure and diversity. Specifically we addressed the following questions: (1) what are the most likely hybrid origins of the polyploid taxa?; (2) are single or multiple origins evident for the polyploid taxa?; (3) is the apomictic breeding system of polyploid taxa obligate or facultative?; (4) what is the source of genetic diversity within and among the polyploid taxa? Finally, we draw on our genetic findings to make robust recommendations for conservation and management of this often rare and complex group of taxa.

## Materials and Methods

### Plant Material

Molecular analysis was carried out on 207 individuals of nine *Sorbus* species collected from 35 sites (Figure 2). Fully expanded disease-free leaf material was collected, dried and stored in silica gel prior to DNA extraction. In addition, 145 trees were re-sampled to provide fresh leaf material for use in flow cytometry. The fresh samples were stored in moist tissue, in polythene bags at 4°C for up to 7 days before use. Voucher specimens were placed in the Welsh National Herbarium, Cardiff (NMW). Each tree had its location described and recorded with a GPS unit. Full details of sample locations, site codes and herbarium accession numbers can be found in Supplementary Table S1.

### DNA Extraction and Molecular Markers

DNA was extracted from dried leaf samples with the Qiagen DNeasy plant mini kit (Qiagen, Hilden, Germany) following the manufacturer’s protocol.

Fifteen previously published microsatellite loci were used; CH01F02, CH01F09, and CH02D11 were developed for use in *Malus* × *domestica* (Gianfranceschi et al., 1998); MSS5, MSS16, and MSS13 for *S. torminalis* (Oddou-Muratorio et al., 2001); SA01, SA19.1, SA03, SA06, SA02, SA08, SA09, and SA14 for *S. aria* (González-González et al., 2010) and MS14 for *Malus* (Nelson-Jones et al., 2002). Primers for four loci: CH01F02, CH01F09, CH02D11, and MSS16; were redesigned by Robertson et al. (2010) for use in a wide range of *Sorbus* taxa. The primers were combined into three multiplexes for amplification; see Supplementary Table S2 for multiplex design. PCR conditions follow that of Hamston et al. (2017).

Capillary electrophoresis of the PCR products was carried out on a Beckman Coulter sequencer (Beckman Coulter, Fullerton, CA, United States) and fragment analysis was performed using CEQ 8000 Genetic Analysis system (Beckman Coulter), followed by a manual verification of each call to ensure proper peak designation. To determine a standard genotype for each polyploid taxon, a reference selection of all study species was subjected to three PCR repeats and used as an internal standard against which all mutations and scoring errors were checked. Any subsequent inconsistent samples were repeated to ensure observed allele sizes were not artifacts of PCR amplification or scoring error.

Thirteen primer pairs successfully amplified across all polyploid taxa; however, primers for three loci (SA19.1, SA02, and SA09) failed to amplify alleles in many *S. aria* and *S. torminalis* individuals (the two putative ancestral diploid taxa). Therefore, analyses that included both diploid and polyploid taxa were based on 10 loci. The additional three loci were included for analysis of polyploids only. Locus MS14 and locus CH01F09 were genome specific for *S. torminalis* and *S. aria*, respectively, and were used to study inheritance patterns and infer the route of formation for those species in subgenus *Tormaria* which are putative hybrids between members of subgenus *Aria* and *S. torminalis.*

### Estimation of Ploidy

Since variation in ploidy within species can indicate different routes of formation and origins, we investigated cytotype diversity within the study species, thereby extending the study of Pellicer et al. (2012). Reliable ploidy allocation for our study species also avoided potential problems arising from unknown or inconsistent ploidy when interpreting microsatellite amplification patterns. We used flow cytometry to estimate nuclear DNA content and to infer ploidy for our samples. Propidium iodide flow cytometry (FCM) analysis was performed as described by Pellicer et al. (2012) at the Jodrell Laboratory (Royal Botanic Gardens, Kew, United Kingdom). Ploidy was inferred by means of the ratio between the target sample peak and that of a known internal standard, *Oryza sativa* [‘IR36’, 2C = 1 pg; Bennett and Smith (1991)]. Standard FCM ratios of several *Sorbus* species analyzed previously (Pellicer et al., 2012) provided an additional baseline against which to compare our samples.

### Data Analyses

Genetic distances between genotypes were calculated by constructing matrices of pairwise Bruvo genetic distances (Bruvo et al., 2004) in the POLYSAT package in R (Clark and Jasieniuk, 2011). Bruvo distances take into account step-wise mutation processes without the requirements of allele copy number and without the requirement that individuals be the same ploidy, thus making the method appropriate for use with mixed ploidy samples (Dufresne et al., 2014). Ploidy information was designated according to the results of FCM analysis. Different sample sets were used to investigate the various aspects of genetic structure, diversity and parentage of our study species.

#### Population Genetic Structure Within *Sorbus*

A prevalence of apomictic reproduction within polyploid taxa can result in clonal groups of genetically identical individuals. Therefore, to infer evolutionary relationships among the study taxa and to determine likely hybrid origins, the samples were assembled into 82 multi-locus genotypes (MLGs) and a pairwise distance matrix was constructed. Patterns of genetic structure among these genotypes were examined using a principal coordinate analysis (PCoA). The results of the PCoA were visualized in 3D using the R package ‘pca3d’ (Weiner, 2015). To summarize the relationships among the groups, the distance matrix was also used to construct a neighbor-joining (NJ) tree using SplitsTree 4 (Huson and Bryant, 2006).

#### Genotypic Diversity

To determine whether the polyploid taxa analyzed had single or multiple origins, to identify the breeding systems prevalent within the group of study species and to identify potential sources of genetic diversity within the polyploid taxa, we analyzed the genotypic diversity within and among our study taxon group.

The following diversity calculations, implemented in POLYSAT, were carried out for the microsatellite data for the 10 loci that amplified successfully in all taxa. As sample size is known to affect allelic diversity statistics (Pruett and Winker, 2008), rarefaction was applied to the diploid species *S. torminalis*, with random sub-sampling of the 33 *S. torminalis* samples to match the sample size of *S. aria* (13 individuals). Total number of different alleles (A) for each species was calculated from the 10 loci that amplified in all taxa. The total number of MLGs (Ng) was determined using the ‘assign clone function’ in POLYSAT with zero as the threshold; thus, all pairs of individuals with a non-zero genetic distance were considered as separate MLGs. Genotypic diversity for each of the polyploid species was determined as the complement of Simpson’s (1949) index λ (1 - λ) (Arnaud-Haond et al., 2007). Simpson’s index gives an unbiased estimator of λ for a given sample size and allows the calculation of a value for genotypic diversity based on the number of MLGs, which varies positively with clonal heterogeneity. The complement of λ (1 - λ) was used to compare genotypic diversity among all species and described the probability of encountering distinct MLGs when taking two units at random from the sample.

To determine whether any of the diversity seen within polyploid taxa could be explained by genetic recombination, either as a result of an interspecific or intraspecific hybridization, or through somatic mutation, we assigned each polyploid sample to a clonal lineage, within which any diversity was considered to be due to mutation using the method of Douhovnikoff and Dodd (2003). In this case, data from the 160 polyploid samples genotyped at 13 microsatellite loci were used. To establish a threshold of genetic distance above which a recombination event would be indicated, frequency histograms for all pairwise genetic distances between samples were plotted. Such histograms are often multi-modal due to highly uneven relative abundance of clones in the data set. The position of the ‘valley’ between the first peak which is close to zero and represents almost identical genotypes (where small differences may be due to somatic mutations or scoring errors in the data set) and the second peak, which represents distinct but closely related clones each deriving from a single reproductive event, is considered an appropriate threshold (Arnaud-Haond et al., 2005). The resulting threshold was then employed to assign all samples to clonal groups using POLYSAT.

#### Parentage Analysis and Genome Inheritance

To assess the most likely hybrid origins for each polyploid species, we matched its allele sizes at 10 loci to those of putative parental species (Robertson et al., 2010). Where all the alleles were present in a pair of putative parents, it was considered a potential hybrid match. All possible pairings were considered in turn and compared to the MLG of each polyploid species. The numbers of alleles missing from the combined MLGs of each putative parent pair was recorded, i.e., a zero score indicated that all alleles were present in a pair of putative parents for that particular species.

The majority genotype was used for each apomictic polyploid species where >80% of individuals had identical MLGs and small allele variations were only observed in the minority. The exception to this was the highly variable locus SA06 where several allele size variants were included for *S. rupicola* and *S. vexans.*

For the sexual parental species, all alleles from all samples were considered together as a pool of potential alleles, since the original genotype was presumed extinct.

To identify the relative contributions of *S. aria* and *S. torminalis* genomes to study members of subgenus *Tormaria*, we used the genome specific loci CH01F09 and MS14, with alleles matched to either *S. torminalis* or *S. aria*. The proposed genome contributions are shown in Figure 1.

#### Flow Cytometry Data

A one-way ANOVA was used to test for differences among the peak ratios of all species, followed by *post hoc* Tukey comparisons of means to determine where any differences lay. The normality of the data distributions was tested using the Shapiro–Wilk test and the homogeneity of variances using Levene’s test. All statistical analysis was performed using R (R Development Core Team, 2015).

## Results

### Microsatellite Markers

Thirteen microsatellite loci were amplified successfully across the seven polyploid species, with 10 of these loci amplifying successfully across all nine study species. The two genome-specific loci, CH01F09 and MS14, yielded alleles for all three members of subgenus *Tormaria*, (*S. subcuneata, S. admonitor*, and *S. devoniensis*) confirming their status as hybrids between subgenus *Aria* and *S. torminalis*.

The 10 core loci (which amplified across all nine study species) yielded a total of 202 alleles from 207 *Sorbus* samples, ranging between eight (MSS13 and SA03) and 24 (SA14) alleles per locus. *S. torminalis* had the highest allelic diversity with 122 alleles recorded in 33 samples (after subsampling, 13 samples yielded 74 alleles at 10 loci). In 161 polyploid samples screened at 13 loci, 123 alleles were amplified, with SA06 yielding the highest number (15 alleles) and SA03 the lowest (four alleles). The allele sizes observed at each locus for each species are given in Supplementary Table S3. The maximum number of alleles per locus per individual corresponded with expected ploidy with the exception of one sample of the normally diploid *S. torminalis*; this particular sample had three alleles at two loci: SA19.1 and SA1 (see the section “Ploidy analysis”).

### Population Genetic Structure and Evolutionary Relationships

The investigation of genetic structure revealed that each of the study species is genetically differentiated, although clustering patterns varied between the two subgenera.

**FIGURE 3:**
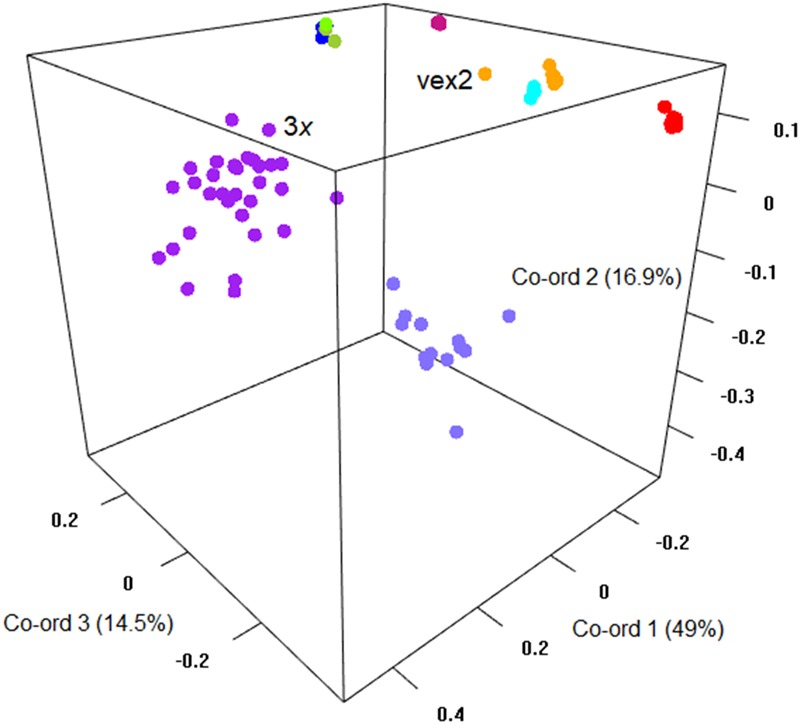
Principal coordinate analysis of the Bruvo distance matrix of 80 MLGs from nine species based on 10 microsatellite loci. Percentages of total variance explained by the co-ordinates are given in parentheses. Currently recognized taxa are indicated as follows: 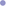 *S. aria*, 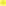 *S. torminalis*, 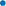 *S. subcuneata*, 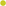 *S. devoniensis*, 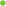 *S. admonitor*, 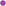 *S. margaretae*, 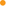 *S. vexans*, 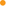 vex2, 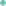 *S. rupicola*, 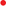 *S. porrigentiformis*.

**FIGURE 4:**
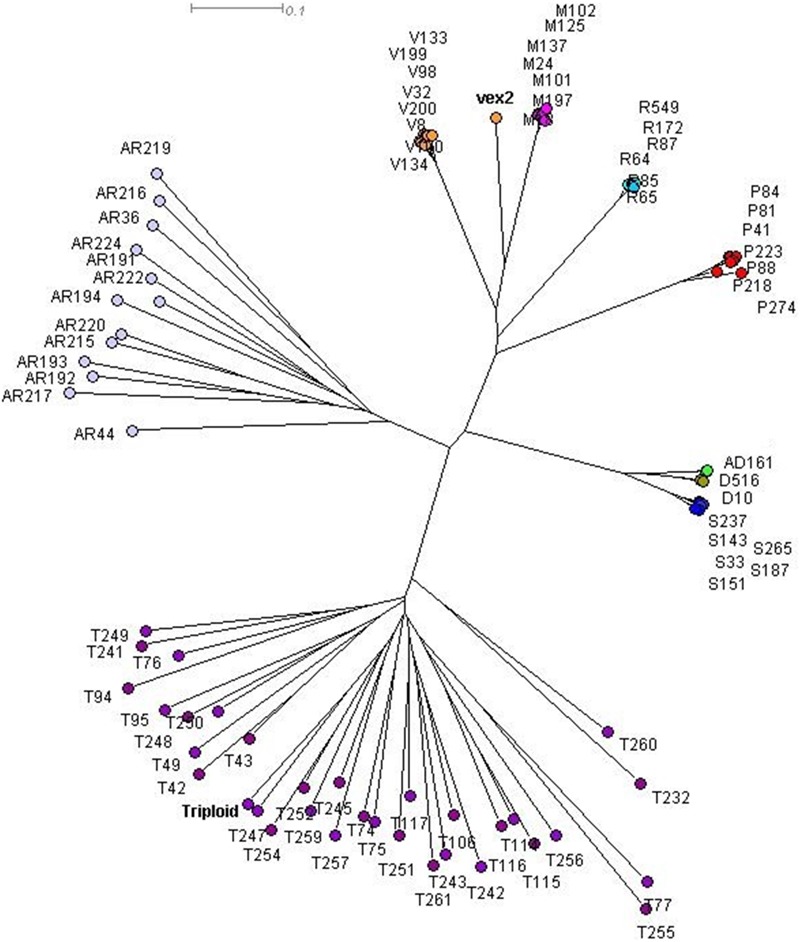
Neighbor-joining (NJ) tree of 80 *Sorbus* MLGs constructed using a Bruvo distance matrix in SplitsTree 4.0. Sample colors correspond to those given in Figure 3. vex2 indicates the second *S. vexans* clone. The triploid *S. torminalis* individual is indicated.

The sexual diploid taxa *S. aria* and *S. torminalis* were differentiated from each other and from all polyploid taxa in the PCoA and NJ tree (Figures 3, 4). Samples from the polyploid taxa fell broadly into two groups corresponding to the two subgenera in the NJ tree. However, the three members of subgenus *Tormaria* (triploid *S. subcuneata*, tetraploid *S. admonitor*, and *S. devoniensis*), were closely grouped in the PCoA, particularly the two tetraploids. Their separation was also weak in the NJ tree where, although distinct clusters are observed, they were positioned on relatively short branches. Individuals of *S. vexans* (4*x*) and *S. rupicola* (4*x*) were also tightly grouped in the PCoA, with the exception of two samples within *S. vexans* that clearly occupied an intermediate position between *S. vexans* and *S. margaretae* (4*x*) and are referred to hereafter as vex2; this intermediate position mirrors that seen in the NJ tree analysis (Figure 4). The *Sorbus margaretae* and *S. porrigentiformis* (4*x*) samples all grouped together in single, highly differentiated clusters in both analyses.

### Genotypic Diversity Within and Among Taxa

The outcrossing diploid species showed high levels of genotypic variation in comparison to the polyploid species that were characterized by few MLGs. The total number of alleles (A), measured across 10 loci, was highest for the diploid, sexual species *S. aria* and *S. torminalis.* In contrast, the polyploid taxa had approximately half the number of alleles, despite their larger genome sizes. Across the 10 loci, no private alleles (alleles present in no other taxa) were observed in any of the seven polyploid taxa, while high levels of allele sharing were noted across all species. Only four of the 95 alleles present in the polyploid taxa were not sampled within the diploid species. The polyploid taxa contained 62% of the total alleles observed. When using a zero threshold to identify unique MLGs (Ng), the number of MLGs observed in the diploids equaled the sample number, as would be expected for sexual, outcrossing taxa. In contrast, low numbers of MLGs were detected within the apomictic polyploid species, although all showed more than one MLG, except *S. admonitor.* The complement of Simpson’s diversity (1 - λ) for the 10 loci also reflected this pattern, with members of subgenus *Tormaria* ranging from 0 (*S. admonitor*) to 0.27 (*S. subcuneata*). *Sorbus margaretae* showed the lowest genotypic diversity in subgenus *Aria*, and *S. vexans s.l.* the highest; diversity statistics are summarized in Table 1.

**Table 1:** Allelic and genotypic diversity found in the studied *Sorbus* species.

Taxon	N	N_i_	X	A	Ng	1 - λ	1 - λ	Nc
						(10 loci)	(13 loci)	
*S. aria*	n/a	13	2	65	13	1	n/a	–
*S. torminalis*	n/a	13	2, 3	74	13	1	n/a	–
*S. admonitor*	c.110	19	4	29	1	0	0	1
*S. devoniensis*	>450	32	4	30	2	0.06	0.17	1
*S. subcuneata*	c.300	27	3	26	4	0.27	0.34	1
*S. margaretae*	c.100	29	4	33	6	0.43	0.48	1
*S. porrigentiformis*	>100	17	4	36	6	0.74	0.78	1
*S. rupicola*	c.40	13	4	35	6	0.79	0.79	1
*S. vexans s.l.*	c.70	24	4	46 (35)	9 (8)	0.86 (0.84)	0.86 (0.84)	2

*N, approximate population size in study region across sampled sites (Rich et al., 2010); N_*i*_, number sampled; X, ploidy from flow cytometry and total number of alleles at any locus; A, total number of alleles observed at 10 loci; Ng, total number of multi-locus genotypes; 1 - λ, Simpson’s complement (Arnaud-Haond et al., 2007) for all species at both 10 and 13 loci with values in parenthesis for *S. vexans s.s.*; Nc, number of clonal linages when a threshold of 0.09 is applied to delineate between sexual and asexual relations between samples (apomictic polyploid species only). Diversity indices for *S. torminalis* were calculated on a sub-sample of 13 of the 33 sampled individuals.*

**FIGURE 5:**
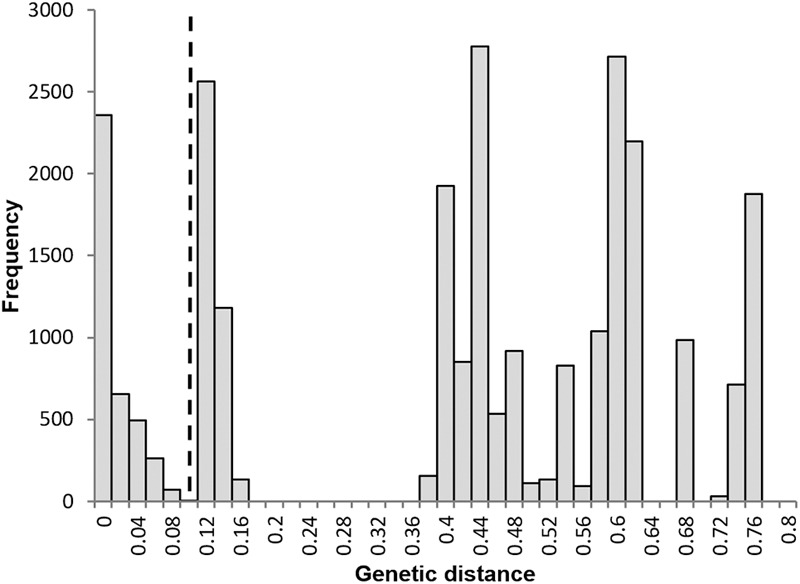
Frequency distribution of pairwise genetic distances between all polyploid individuals. *N* = 160. No. of pairwise comparisons = 25,600. The dashed line represents the threshold distance applied to separate asexually and sexually related individuals = 0.09.

A frequency histogram of all pairwise distances was multi-modal (Figure 5), with a clear peak at zero indicating the prevalence of identical genotypes and indicating an apomictic mode of reproduction. The threshold distance between the first and second peak was 0.09; this value was used to assign all polyploid genotypes to a clonal lineage. Each polyploid species corresponded to a single clonal lineage with the exception of *S. vexans* that had two different clones, each of which conformed to a distinct genotype; these two genotypes differed at all 13 loci analyzed. This result is inconsistent with only mutational variation and suggests a separate sexual origin for each *S. vexans* clone; vex2 formed the second clone, consisting of two identical samples. Diversity statistics for *S. vexans* were calculated with and without vex2, as *S. vexans s.l.* and *S. vexans s.s.*, respectively.

### Parentage

Locus MS14 amplified only in *S. torminalis* and members of subgenus *Tormaria*, with two alleles amplified for each of the tetraploid species *S. devoniensis* and *S. admonitor*, and one allele for the triploid *S. subcuneata.* Two alleles were amplified at locus CH01F09 for these three species, indicating an even contribution of alleles from *S. aria* and *S. torminalis* for the tetraploids and a 2:1 ratio of alleles for *S. subcuneata*, respectively. *S. subcuneata, S. devoniensis* and *S. admonitor* shared alleles with each other and diploid *S. torminalis* at 10 loci. For these three polyploid species, pairwise comparisons (of putative parental pairs) generated multiple genotype matches, including a match of S. *margaretae* × *S. torminalis.*

The tetraploid members of subgenus *Aria* all had four alleles at the *Aria* genome-specific locus CH01F09 and no alleles at locus MS14. Thus, *S. torminalis* and members of subgenus *Tormaria* cannot be considered as potential parents of any member of subgenus *Aria* studied. Only *S. margaretae* and the second *S. vexans* clone (vex2) had alleles matching those of any other taxa studied with 10 loci. Pairwise comparisons identified no exact matches indicative of potential parentage between members of subgenus *Aria* and the taxa studied. For partial parentage matches, all missing alleles were subsequently checked against the minority genotypes for each potential (unresolved) progenitor. Consequently, the next best match was recorded with a score for the number of missing allele matches against each pairwise cross (Table 2).

**Table 2:** Summary of the parentage simulation.

Taxon	Parent pair X		Number of mismatched alleles
*S. subcuneata*	*S. admonitor*	*S. devoniensis*	0
	*S. admonitor*	*S. margaretae*	0
	*S. admonitor*	*S. vexans*	0
	*S. admonitor*	*S. porrigentiformis*	0
	*S. admonitor*	*S. rupicola*	0
	*S. admonitor*	*S. torminalis*	0
	*S. admonitor*	*S. aria*	0
	***S. torminalis***	***S. margaretae***	0
*S. admonitor*	*S. torminalis*	*S. devoniensis*	0
	***S. torminalis***	***S. subcuneata***	0
	*S. torminalis*	*S. margaretae*	0
*S. devoniensis*	*S. torminalis*	*S. devoniensis*	0
	***S. torminalis***	***S. subcuneata***	0
	*S. torminalis*	*S. margaretae*	0
*S. margaretae*	*S. aria*	*S. rupicola*	4
	***S. vexans***	***S. rupicola***	5
*S. vexans*	*S. margaretae*	*S. porrigentiformis*	5
	***S. aria***	***S. rupicola***	6
	*S. margaretae*	*S. rupicola*	6
	*S. margaretae*	*S. aria*	6
vex2	***S. vexans***	***S. margaretae***	6
*S. porrigentiformis*	***S. aria***	***S. rupicola***	13
	*S. aria*	*S. vexans*	13

*For each polyploid taxon the best-matched pairs of potential parent genotypes are shown. The best matches have the lowest number of mismatched or missing alleles as shown in the right column. Based on the results from all analyses the most likely pairings are shown in bold.*

### Ploidy Analysis

It was possible to allocate ploidies for 145 samples of eight species. The flow cytometric analyses revealed three cytotypes: diploid (2*x*), triploid (3*x*) and tetraploid (4*x*). The *Sorbus* samples and the internal size standard (*Oryza sativa*) produced clear histogram peaks with low coefficients of variation (Table 3, CV %: 1.91–3.74; mean = 2.54 ± 0.39).

**Table 3:** Nuclear DNA content (pg) of each of the clusters with inferred ploidy and chromosome number (2*n*).

Ploidy	2*n*	N	Relative 2C DNA (pg)		CV (%)
			Min – max (pg)	Mean ± SD	
2x (*S. torminalis*)	34	26	1.600 – 1.678	1.63 ± 0.018	3.25
3x (*S. torminalis*)	51	1	2.38	2.38	2.98
3x (*S. subcuneata*)	51	31	2.286 – 2.386	2.33 ± 0.022	2.52
4*x*	68	87	2.857 – 3.231	3.07 ± 0.084	2.53

*The triploid *S. torminalis* is shown separately. N, sample size; CV, coefficient of variation.*

#### Determination of Ploidy Levels

The ratios between the sample peaks and internal size were used to infer the ploidy of each sample based on previous results for *Sorbus* (Pellicer et al., 2012). Comparison of the mean peak ratios confirmed that each of the three ploidies was of a significantly different size [Supplementary Figure S1; one-way analysis of means (not assuming equal variances): *F* = 15132, *df* = 2, *n* = 145, *p* < 2.2 × 10^-16^]. Apomictic taxa were all polyploid. One *S. torminalis* sample showed three alleles at two loci and had an increased nuclear DNA content, and was confirmed as a triploid (see Hamston et al., 2017). *S. subcuneata* was also confirmed as triploid, as all samples had a nuclear DNA content consistent with a triploid cytotype (see Table 3). The remaining six polyploid species were all confirmed as tetraploid. However, the tetraploid species within subgenus *Tormaria* (*S. admonitor* and *S. devoniensis*) displayed significantly larger genome sizes than those of the four subgenus *Aria* tetraploids (Supplementary Figure S1; ANOVA: *F* = 16.738, *n* = 87, *p* ≤ 0.001).

## Discussion

Our investigation of relationships among often sympatric populations of polyploid *Sorbus* species revealed that each currently described species is readily genetically differentiated. In contrast to other studies (e.g., Robertson et al., 2010), we found that all polyploid species studied, with the exception of *S. admonitor*, exhibited multiple MLGs. Each MLG was composed largely of alleles in common with other taxa, resulting in high levels of apparent allele sharing across all study species. Significantly, our results support the hypothesis that the route of polyploid formation in *Sorbus* is via interspecific hybridization. However, whilst the genetic integrity of each polyploid taxon is largely maintained via apomixis, genetic mutation has led to ‘clone mates’ within each of the described species.

### Breeding System and Genotypic Variation in Sorbus

The patterns of genetic diversity within and among our study taxa appear to be a consequence of breeding system and mutational load. Our results demonstrate that the polyploid *Sorbus* populations analyzed are predominantly apomictic, in accordance with the findings of a previous isoenzyme-based study (Proctor et al., 1989). This is evident in the low levels of genetic variability detected within each polyploid species, in contrast to the sexual diploids *S. aria* and *S. torminalis* which were highly variable, having a unique MLG for each sample (Table 1), as would be expected for self-incompatible, outcrossing species. Sexual reproduction appears to be a rare event within the polyploid taxa – we were unable to find evidence of it within the individuals we sampled. However, if sexual reproduction does occur within these largely apomictic species, it would only be revealed if parental alleles were absent in the resulting offspring. Because plants of the same clone produce characteristically few allelic combinations of gametes, offspring produced sexually may have the same MLG as the parental individuals and, therefore, would not be readily distinguishable.

We also sampled established trees, which must have resulted from viable seeds and successful seedlings. Therefore, it may be that the apomictic clones sampled were those best adapted to their environment, with –ergo– other genetic combinations being less viable.

If we accept that the principal route of polyploid formation in *Sorbus* is via hybridization involving a diploid parental species and a facultative apomict (Robertson et al., 2004, 2010; Hajrudinović et al., 2015b), the rate of novel polyploid formation will depend on the abundance and relative distributions of the parental taxa and to what degree apomixis is facultative. The sexual diploids, *S. torminalis* and *S. aria*, currently co-occur only rarely with any of the endemic polyploid taxa studied here, so current opportunities for hybridization between diploid and polyploid taxa appear to be rare.

### Source of Genetic Variability Within Apomicts

Our study has revealed genetic variability in six of the seven apomictic polyploid taxa. Although the levels of variation were low, polymorphisms at a number of loci were sufficient to identify divergent ‘clone mates’ associated with particular sites. In the absence of recombination events, mutation plays a key role in the generation of genetic variation in apomictic lineages (Paun et al., 2006). *Sorbus subcuneata, S. rupicola, S. porrigentiformis, S. margaretae*, and *S. vexans* all showed small numbers of site-associated mutations. One of these mutational variants (*S. subcuneata* from Greenaleigh Wood, near Minehead, Somerset; Figure 2 and Supplementary Table S1) had been identified previously as having some variation in leaf morphology compared to those at other sites (T.C.G. Rich, *pers. comm*.). If so, this could suggest a greater level of phenotypic variation than that detected with the microsatellite loci used in this study. The small site of Neck Wood (north Devon, Figure 2 and Supplementary Table S1) was associated with specific clonal variants for *S. margaretae* and *S. rupicola* at loci SA06 and SA09. However, wider interpretation of spatial patterns evident using the SA06 locus should be cautioned against since there is high likelihood of allele size homoplasy due to combinations of expansion and contraction of the microsatellite repeat motif in different lineages, a feature linked to high mutation rates, particularly of dinucleotide repeats (Ellegren, 2000a). Nonetheless, the use of more variable markers may reveal spatial patterns that relate to possible colonization routes, a potentially interesting line of investigation. The variation seen previously in isozyme banding patterns in *S. margaretae* at the western end of its distribution (Proctor et al., 1989) could correspond with some of the site-specific mutational variation associated with *S. margaretae* at Neck Wood (the most western location recorded for this species) or could indeed be the vex2 variant, also found at Neck Wood.

Members of subgenus *Tormaria* showed little mutational variation compared to subgenus *Aria.* Such a finding suggests they may be of more recent origin than the members of subgenus *Aria*, particularly *S. admonitor* that has the most restricted distribution of all the species included in the current study. Indeed, the genetic variation within the apomictic taxa was in marked contrast to that found in similar taxa from the Avon Gorge, which showed consistently invariable genotypes using the same genetic markers (Robertson et al., 2010). This lack of variation suggests that the Avon Gorge endemic species may be of more recent origin. Additionally, the wider geographic distribution of the majority of the species analyzed in the current study suggests they maybe older than the Avon Gorge endemic species. Likewise, perhaps due to mutations in the primer sites accrued over time, time since speciation may explain why three loci that amplified in the polyploids failed to amplify in many of the diploid samples.

### Relationships Among Polyploid and Diploid Taxa

**FIGURE 6:**
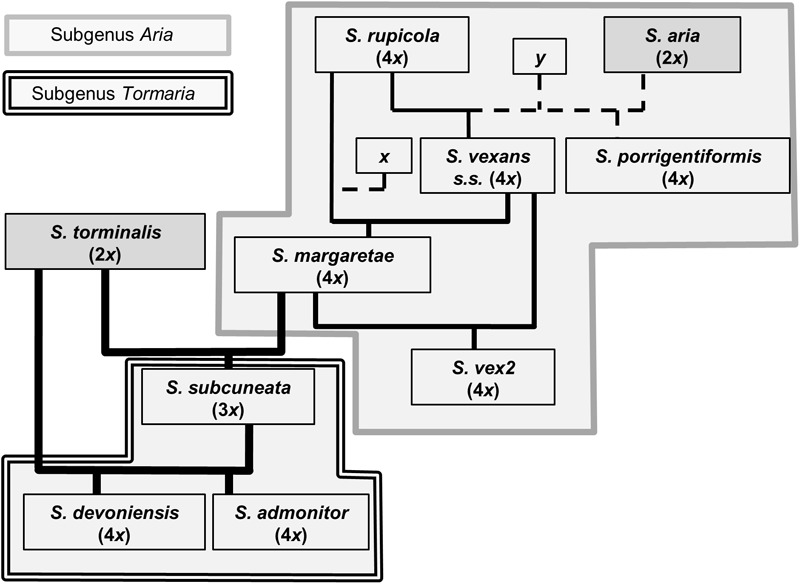
Proposed relationships among *Sorbus* species sampled from southwest Britain. Ploidy levels, as determined by FCM, are given in parentheses. Line weight indicates approximate strength of supporting evidence. Dashed lines indicate speculative relationships and ‘*x*’ and ‘*y*’ indicate possible missing intermediate genotypes or indirect origins.

The use of nuclear microsatellite markers has enabled the hypothesized evolutionary relationships shown in Figure 1 to be refined. Using the data from this study, we were able to postulate detailed evolutionary relationships and likely hybrid origins among our study taxa (summarized in Figure 6). The patterns of introgression observed within the study taxa suggest that co-existence of many of these species may have persisted over long time periods.

Discrete clusters of samples in the PCoA and NJ tree (Figures 3, 4) correspond to the nine currently recognized species. The close relationships among polyploid species indicate probable linkage through ancestral hybrid events or on-going gene flow. Shared hybrid origins are the most likely reason for the many shared alleles observed, with frequent gene flow an unlikely explanation due to the predominance of apomixis among the study polyploid taxa.

The presence of several alleles shared in the endemic polyploids that were not found among the diploids (*S. aria* and *S. torminalis*), *S. porrigentiformis* or *S. rupicola* is strongly suggestive of missing intermediate parental genotypes (denoted as *x* in Figure 6). One of these alleles was matched to *S. rupicoloides*, an endemic of Cheddar Gorge, Somerset (see Figure 2), sampled by Houston et al. (2009). This illustrates the close relationships among regional polyploids and may indicate colonization routes for the more widespread polyploid *Sorbus* in southwest Britain. Our data suggest that hybridization among polyploid *Sorbus* has been a route of further polyploid formation. This is supported by the high level of allele sharing among the polyploids, the fact that three microsatellite loci failed to amplify in the majority of *S. aria* and *S. torminalis* (indicating a more distant relationship), and the absence of either *S. aria* or *S. torminalis* at many of the study sites, although the latter may not always have been the case, as discussed below.

Our analyses confirm the accepted view that members of subgenus *Tormaria* (*S. subcuneata, S. admonitor* and *S. devoniensis*) are distinct from –but intermediate to– *S. torminalis* and subgenus *Aria*, in line with the hybrid origins proposed by Nelson-Jones et al. (2002). The larger genome size of *S. admonitor* and *S. devoniensis*, when compared to the tetraploid members of subgenus *Aria*, adds weight to their probable hybrid origin, with *S. torminalis* as an ancestral parent (Chester et al., 2007), since *S. torminalis* has the largest genome size of the three diploid *Sorbus* species tested by Pellicer et al. (2012). The intermediate position of these members of subgenus *Tormaria* between *S. torminalis* and tetraploid *S. margaretae* (subgenus *Aria*) in the PCoA suggested *S. margaretae* may be the male ancestral parent of these representatives of subgenus *Tormaria*, rather than *S. aria* or *S. rupicola*, as proposed by Sell (1989). This relationship would explain the many shared alleles among *S. margaretae, S. devoniensis, S. admonitor* and *S. subcuneata* and is in accordance with results from the pairwise matches in Table 2. Subgenus *Tormaria* formed a tight group of very closely related taxa in all our cluster analyses. *Sorbus subcuneata*, a triploid taxon, shared all its alleles with both tetraploids, *S. devoniensis* and *S. admonitor*, across all 13 loci, implying a common recent origin for the group, possibly with *S. subcuneata* as an ancestral species for *S. admonitor* and *S. devoniensis*. The additional alleles present in both tetraploids were also found in some of our samples of *S. torminalis* (Supplementary Table S3). Thus, both *S. admonitor* and *S. devoniensis* were likely formed from repeated hybrid events between *S. subcuneata* and *S. torminalis* (Supplementary Figure S2). This poses the question as to whether, taxonomically, these should be considered variants of the same species with multiple origins as suggested by Proctor et al. (1989) and Sell (1989), as per *S. arranensis* in Scotland (Robertson et al., 2004). Certainly, the geographical distribution of triploid *S. subcuneata* overlaps with *S. admonitor* and *S. devoniensis*, but the spatial separation of the latter two suggests they may have originally arisen in different locations.

Firstly, these relationships confirm the role of triploids in tetraploid formation via the ‘triploid bridge,’ which is consistent with the formation of tetraploid *Sorbus* elsewhere (Robertson et al., 2004, 2010; Hajrudinović et al., 2015b). Since some apomictic triploid *Sorbus* taxa still produce some viable pollen (Rich, 2009) it is most likely that *S. subcuneata* was the male parent in these hybrid events. Secondly, the formation of triploid *S. subcuneata* via fertilization of a reduced diploid egg with reduced tetraploid pollen confirms this as a potentially key route of tetraploid formation, in line with recent studies by Ludwig et al. (2013). Therefore, despite current distribution patterns, historically, *S. torminalis* must have occurred along the north coastal areas of our study region in sympatry with *S. subcuneata*. Currently, it co-occurs with *S. devoniensis* at a number of sites, which may indicate similar ecological requirements; *S. devoniensis* is found on a wider range of geologies and soil types than other polyploid species (Rich et al., 2010). However, historical human-aided dispersal of *S. torminalis* cannot be ruled out, since they are largely found as hedgerow trees.

Whilst diploid *S. torminalis* appears to be a driving force for the generation of members of subgenus *Tormaria*, our data also show that occasional hybridization between polyploids, most probably tetraploids, may also be responsible for the generation of novel genotypes. The relationships among tetraploids *S. rupicola, S. vexans, S. margaretae* and vex2, suggest repeated hybridization and introgression within this group of apomicts. Within subgenus *Aria, S. rupicola* is the most likely parental polyploid species for endemics *S. vexans* and *S. margaretae*, either directly or indirectly, rather than *S. porrigentiformis.* Although they both occur across the range of the local endemic taxa within our group, the PCoA shows *S. rupicola* is more closely related to other members of subgenus *Aria* in our study group; this is in contrast to other sites in southwest Britain where *S. porrigentiformis* is thought to be one of the primary parental taxa, hybridizing with diploid *S. aria s.s.* (Houston et al., 2009; Robertson et al., 2010).

Both *S. vexans* and *S. margaretae* are endemic to this region and in the absence of *S. aria* their origin could be via allotetraploid hybridization rather than a diploid × polyploid cross. The close affinity of *S. vexans s.s*. to *S. rupicola* rather than *S. aria* in the PCoA analysis, and the sharing of many alleles, suggests *S. vexans s.s*. is derived from *S. rupicola*, possibly with *S. aria* as the other parent, but the lack of exact parental matches indicates possible missing intermediate genotype(s) (denoted as *y* in Figure 6) that are now either extinct or existing as cryptic hybrids within the current *Sorbus* distributions. These may include once native *S. aria* or extinct triploids acting as the bridge between diploids and tetraploid formation. Triploid *Sorbus*, in common with other triploids in Rosaceae, are less fertile than diploids and tetraploids (Talent and Dickinson, 2005; Ludwig et al., 2013) so they may occupy a more transient evolutionary position.

Our results suggest that *S. margaretae* may have a more recent origin than *S. vexans s.s.* Their relative genotypic variation due to mutation (1 - λ values: *S. margaretae*, 0.43 vs. *S. vexans s.s.*, 0.84) could indicate a more recent origin, since mutations accrue over time since divergence (Ellegren, 2000b). Moreover, they are closely related with very similar leaf morphologies, being hard to distinguish in the field. However, they are genetically distinct and our clonal analysis attributes their genetic differences to sexual reproduction rather than genetic mutation. Thus, the most parsimonious hybrid origin of *S. margaretae* is *S. vexans s.s.* × *S. rupicola*, rather than the closest parental match of *S. rupicola* × *S. aria* (four mismatches, Table 2).

The second *S. vexans* clone (vex2) represents a separate genotype resulting from interspecific hybridization rather than sexual reproduction, since alleles common to other taxa were identified. The intermediate position of vex2 between *S. vexans* and *S. margaretae* in the PCoA, suggests it may be a hybrid involving these two tetraploids, especially since the ancestral diploid progenitor for subgenus *Aria, S. aria*, is not present in the locality. This origin is also supported by the pairwise matching (Table 2). vex2 occurs at Neck Wood, a small (<3 ha) coastal site (Figure 2), which has a high diversity of polyploid *Sorbus* species: *S. subcuneata, S. devoniensis, S. margaretae*, S. *rupicola* and *S. vexans*, and specimens of *S. intermedia* (Ehrh) Pers., a non-native that has become naturalized. The vex2 variant is genetically unique and its possible derivation from polyploid taxa in the absence of parental diploid forms indicates that this is a possible route for polyploid *Sorbus* formation and provides strong evidence of on-going diversification via introgression in the region. This finding also raises the likelihood that there may be other cryptic taxa on sites in the region with multiple *Sorbus* taxa where heterospecific pollen pressure is high, further increasing the chances of interspecific hybridization (Hajrudinović et al., 2015b).

*Sorbus porrigentiformis* shares alleles with *S. rupicola* at 11 of the 13 amplified loci, so it seems likely that *S. porrigentiformis* is also derived from *S. rupicola*, maybe indirectly (denoted as *y* in Figure 6), in line with proposed theories (Rich et al., 2010). However, as with previous studies (Robertson et al., 2010), their relationship remains unclear.

The sexually reproducing *S. aria* and *S. torminalis* are clearly differentiated from each other and from the polyploid taxa in all our cluster analyses. The triploid form of *S. torminalis* clusters with its diploid forms (Figures 3, 4), suggesting its origin is due to intraspecific rather than interspecific hybridization. It has been proposed that such cryptic autopolyploids, often form via the fusion of unreduced gametes (Ramsey and Schemske, 1998) and are a more common and important component of plant diversity than perhaps historic views suggest (Soltis et al., 2007). Indeed, Pellicer et al. (2012) identified a number of polyploid *S. aria* samples. However, the triploid *S. torminalis* was found located close to tetraploid *S. devoniensis* (Hamston et al., 2015); thus, the fusion of gametes from diploid and polyploid *Sorbus* cannot be ruled out since they share many alleles. Indeed, wide-scale screening of *Sorbus* seed ploidy has shown this to be the most likely scenario (Hajrudinović et al., 2015b).

### Conservation

Strategies developed for the conservation of polyploid complexes that contain threatened species need to encompass any local adaptations of particular groups, together with the long-term ability of the complex to evolve through natural selection in a changing environment. There should be some assessment of the status of parental taxa, however, common, to ensure they are protected from detrimental human activities. A process-based approach to conservation measures (*sensu* Ennos et al., 2012) should be targeted at locations containing many constituents and putative constituents of species complexes to maximize the potential for further hybridization. In this study, our data have given insight into past hybridization events, in particular the role of *S. torminalis* in the formation of members of subgenus *Tormaria*. At this time, *S. torminalis* is not of conservation concern, however, this research demonstrates why it should be included in any actions to conserve the complex genetic mosaic characteristic of this species group. The formation routes of the constituent members of subgenus *Aria* are less clear, most likely due to a combination of mutation and missing or unsampled parental genotypes. Both these factors indicate that genetic diversity in this group may be higher than our sampling suggests and that process-based conservation action plans should focus on sites of high species diversity –for example, Neck Wood– in order to preserve current *Sorbus* diversity in this region of southwest England.

## Author Contributions

TH carried out the fieldwork. TH carried out the molecular work and statistical analysis under the supervision of RK, NdV, JC, and JS. The flow cytometry was performed by JP, assisted by TH under the supervision of MF. TH and JS drafted the manuscript, and all authors contributed to the final version.

## Conflict of Interest Statement

The authors declare that the research was conducted in the absence of any commercial or financial relationships that could be construed as a potential conflict of interest.
