# Supplementary material for: Apomixis and Hybridization Drives Reticulate Evolution and Phyletic Differentiation in Sorbus L.: Implications for Conservation
Source: Front Plant Sci. 2018 Dec 13;9:1796. doi: 10.3389/fpls.2018.01796 (PMC6300497; doi:10.3389/fpls.2018.01796)
Supplement: Supplementary file 1 [file Data_Sheet_1.docx]

***Supplementary Material***

**Apomixis and hybridisation drives reticulate evolution and phyletic differentiation in *Sorbus* L.: Implications for conservation**

Tracey J. Hamston*^1, 2^, Natasha de Vere^3^, R. A. King^1^, J. Pellicer^4^, M. F. Fay^4, 5^, James E. Cresswell^1^ and Jamie R. Stevens^1^

*** Correspondence:**

Dr Tracey Hamston [tracey.hamston@wwct.org.uk](mailto:tracey.hamston@wwct.org.uk)

Dr Jamie Stevens: [j.r.stevens@exeter.ac.uk](mailto:j.r.stevens@exeter.ac.uk)

**Supplementary Data**

**Table S1.** Location details for all DNA samples. Site codes link to map (Fig. 2). Luckbarrow is a collection held by the Exmoor Natural History Society at Porlock (51.203371 lat. -3.5844902 long.)

| **Sample no:** | **Accession no:** | **Taxon** | **Lat.** | **Long.** | **Site** | **Site code** |
| --- | --- | --- | --- | --- | --- | --- |
| 5 | V.2014.003.143 | *S. admonitor* | 51.22675 | -3.79965 | Watersmeet | WM |
| 6 | V.2014.003.142 | *S. admonitor* | 51.22501 | -3.79907 | Watersmeet | WM |
| 56 | V.2014.003.140 | *S. admonitor* | 51.22467 | -3.80003 | Watersmeet | WM |
| 57 | V.2014.003.141 | *S. admonitor* | 51.22494 | -3.80024 | Watersmeet | WM |
| 161 | V.2014.003.171 | *S. admonitor* | 51.22509 | -3.80029 | Watersmeet | WM |
| 162 | V.2014.003.181 | *S. admonitor* | 51.22474 | -3.80033 | Watersmeet | WM |
| 163 | V.2014.003.168 | *S. admonitor* | 51.22469 | -3.80019 | Watersmeet | WM |
| 164 | V.2014.003.182 | *S. admonitor* | 51.22443 | -3.80045 | Watersmeet | WM |
| 168 | V.2014.003.166 | *S. admonitor* | 51.22477 | -3.80015 | Watersmeet | WM |
| 169 | V.2014.003.167 | *S. admonitor* | 51.22459 | -3.80014 | Watersmeet | WM |
| 170 | V.2014.003.169 | *S. admonitor* | 51.22661 | -3.79892 | Watersmeet | WM |
| 171 | V.2014.003.170 | *S. admonitor* | 51.22456 | -3.79831 | Watersmeet | WM |
| 174 |  | *S. admonitor* | 51.22451 | -3.79974 | Watersmeet | WM |
| 178 | V.2014.003.162 | *S. admonitor* | 51.22301 | -3.79608 | Watersmeet | WM |
| 180 | V.2014.003.164 | *S. admonitor* | 51.22436 | -3.79841 | Watersmeet | WM |
| 183 | V.2014.003.165 | *S. admonitor* | 51.22504 | -3.80014 | Watersmeet | WM |
| 214 | V.2014.003.180 | *S. admonitor* | 51.22669 | -3.79968 | Watersmeet | WM |
| 234 |  | *S. admonitor* | 51.22681 | -3.79968 | Watersmeet | WM |
| 553 | V.2014.003.163 | *S. admonitor* | 51.22451 | -3.80147 | Watersmeet | WM |
| 191 |  | *S. aria* | 51.46904 | -2.62975 | Avon Gorge | AG |
| 192 |  | *S. aria* | 51.46867 | -2.63051 | Avon Gorge | AG |
| 193 |  | *S. aria* | 51.46867 | -2.63051 | Avon Gorge | AG |
| 194 |  | *S. aria* | 51.46955 | -2.63484 | Avon Gorge | AG |
| 215 | V.2014.003.175 | *S. aria* | 51.2879 | -2.74631 | Cheddar gorge | CG |
| 216 | V.2014.003.176 | *S. aria* | 51.2879 | -2.75157 | Cheddar gorge | CG |
| 217 | V.2014.003.173 | *S. aria* | 51.28788 | -2.75276 | Cheddar gorge | CG |
| 219 | V.2014.003.179 | *S. aria* | 51.28812 | -2.75229 | Cheddar gorge | CG |
| 220 | V.2014.003.174 | *S. aria* | 51.28674 | -2.75364 | Cheddar gorge | CG |
| 222 | V.2014.003.177 | *S. aria* | 51.2867 | -2.75541 | Cheddar gorge | CG |
| 224 | V.2014.003.172 | *S. aria* | 51.28748 | -2.75555 | Cheddar gorge | CG |
| 36 | V.2014.003.178 | *S. aria* | 51.45552 | -2.63963 | Leigh woods | LW |
| 44 |  | *S. aria* | 51.4661 | -2.63564 | Leigh woods | LW |
| 105 | V.2014.003.096 | *S. devoniensis* | 50.9119 | -4.07305 | Beaford |  |
| 246 | V.2014.003.016 | *S. devoniensis* | 50.8323 | -4.13558 | Highampton |  |
| 103 | V.2014.003.093 | *S. devoniensis* | 50.88709 | -3.98226 | Hollocombe |  |
| 78 | V.2014.003.014 | *S. devoniensis* | 50.77751 | -4.02974 | Inwardleigh |  |
| 79 | V.2014.003.011 | *S. devoniensis* | 50.77758 | -4.02977 | Inwardleigh |  |
| 80 | V.2014.003.008 | *S. devoniensis* | 50.77732 | -4.02965 | Inwardleigh |  |
| 51 | V.2014.003.017 | *S. devoniensis* | 50.56476 | -3.52589 | Little Haldon | LH |
| 52 | V.2014.003.018 | *S. devoniensis* | 50.56479 | -3.52554 | Little Haldon | LH |
| 89 | V.2014.003.006 | *S. devoniensis* | 50.56573 | -3.5364 | Little Haldon | LH |
| 90 | V.2014.003.003 | *S. devoniensis* | 50.56619 | -3.53573 | Little Haldon | LH |
| 91 | V.2014.003.007 | *S. devoniensis* | 50.56491 | -3.53645 | Little Haldon | LH |
| 92 | V.2014.003.004 | *S. devoniensis* | 50.56546 | -3.534 | Little Haldon | LH |
| 93 | V.2014.003.005 | *S. devoniensis* | 50.56544 | -3.53406 | Little Haldon | LH |
| 184 | V.2014.003.084 | *S. devoniensis* | 50.11504 | -3.52031 | Little Haldon | LH |
| 228 | V.2014.003.083 | *S. devoniensis* |  |  | Little Haldon | LH |
| 229 | V.2014.003.086 | *S. devoniensis* | 50.56541 | -3.5341 | Little Haldon | LH |
| 230 |  | *S. devoniensis* |  |  | Little Haldon | LH |
| 231 |  | *S. devoniensis* |  |  | Little Haldon | LH |
| 110 | V.2014.003.085 | *S. devoniensis* | 50.92121 | -4.1573 | Little Torrington |  |
| 516 |  | *S. devoniensis* |  |  | Luckbarrow |  |
| 517 |  | *S. devoniensis* |  |  | Luckbarrow |  |
| 19 | V.2014.003.073 | *S. devoniensis* | 51.21864 | -3.95849 | Neck wood | NW |
| 70 | V.2014.003.009 | *S. devoniensis* | 50.73087 | -3.8987 | South Tawton |  |
| 71 | V.2014.003.012 | *S. devoniensis* | 50.72905 | -3.90449 | South Tawton |  |
| 72 | V.2014.003.010 | *S. devoniensis* | 50.73076 | -3.89058 | South Tawton |  |
| 73 | V.2014.003.015 | *S. devoniensis* | 50.73155 | -3.89127 | South Tawton |  |
| 118 | V.2014.003.094 | *S. devoniensis* | 51.00979 | -4.20639 | Upcott |  |
| 113 | V.2014.003.089 | *S. devoniensis* | 50.93744 | -4.18177 | Watergate Bridge |  |
| 10 | V.2014.003.070 | *S. devoniensis* | 51.22129 | -3.89988 | Woody Bay | WB |
| 11 | V.2014.003.071 | *S. devoniensis* | 51.22084 | -3.89829 | Woody Bay | WB |
| 15 | V.2014.003.072 | *S. devoniensis* | 51.22832 | -3.90799 | Woody Bay | WB |
| 101 |  | *S. margaretae* | 51.22463 | -3.66655 | Culbone | CB |
| 102 |  | *S. margaretae* | 51.22416 | -3.66752 | Culbone | CB |
| 197 |  | *S. margaretae* | 51.22473 | -3.66602 | Culbone | CB |
| 198 |  | *S. margaretae* | 51.22471 | -3.66685 | Culbone | CB |
| 204 |  | *S. margaretae* | 51.22501 | -3.66679 | Culbone | CB |
| 221 |  | *S. margaretae* | 51.22450 | -3.667753 | Culbone | CB |
| 26 | V.2014.003.066 | *S. margaretae* | 51.2337 | -3.7434 | Desolation | DL |
| 27 | V.2014.003.065 | *S. margaretae* | 51.23388 | -3.74425 | Desolation | DL |
| 29 | V.2014.003.082 | *S. margaretae* | 51.23509 | -3.75604 | Desolation | DL |
| 30 |  | *S. margaretae* | 51.23509 | -3.75604 | Desolation | DL |
| 31 | V.2014.003.126 | *S. margaretae* | 51.23509 | -3.75604 | Desolation | DL |
| 35 | V.2014.003.067 | *S. margaretae* | 51.22761 | -3.73556 | Desolation | DL |
| 54 |  | *S. margaretae* | 51.23509 | -3.75604 | Desolation | DL |
| 122 |  | *S. margaretae* | 51.23516 | -3.75698 | Desolation | DL |
| 123 |  | *S. margaretae* | 51.23511 | -3.7567 | Desolation | DL |
| 124 |  | *S. margaretae* | 51.23547 | -3.75818 | Desolation | DL |
| 125 |  | *S. margaretae* | 51.23522 | -3.75512 | Desolation | DL |
| 126 |  | *S. margaretae* | 51.2353 | -3.75522 | Desolation | DL |
| 127 |  | *S. margaretae* | 51.2352 | -3.7553 | Desolation | DL |
| 128 |  | *S. margaretae* | 51.23521 | -3.75532 | Desolation | DL |
| 129 |  | *S. margaretae* | 51.23544 | -3.75565 | Desolation | DL |
| 130 |  | *S. margaretae* | 51.2352 | -3.75586 | Desolation | DL |
| 131 |  | *S. margaretae* | 51.23509 | -3.75604 | Desolation | DL |
| 132 |  | *S. margaretae* | 51.23509 | -3.75604 | Desolation | DL |
| 137 |  | *S. margaretae* | 51.22913 | -3.6964 | Embelle woods | CB |
| 18 | V.2014.003.146 | *S. margaretae* | 51.21869 | -3.95807 | Neck wood | NW |
| 24 | V.2014.003.068 | *S. margaretae* | 51.2193 | -3.95883 | Neck wood | NW |
| 25 | V.2014.003.069 | *S. margaretae* | 51.21927 | -3.95887 | Neck wood | NW |
| 3 | V.2014.003.062 | *S. margaretae* | 51.22528 | -3.79756 | Watersmeet | WM |
| 53 |  | *S. porrigentiformis* | 50.48037 | -3.51315 | Babbacombe slopes | TB |
| 86 | V.2014.003.152 | *S. porrigentiformis* | 50.47946 | -3.51364 | Babbacombe slopes, Torbay | TB |
| 88 | V.2014.003.150 | *S. porrigentiformis* | 50.47946 | -3.51364 | Babbacombe slopes, Torbay | TB |
| 218 | V.2014.003.151 | *S. porrigentiformis* | 51.28799 | -2.7524 | Cheddar gorge | CG |
| 223 | V.2014.003.158 | *S. porrigentiformis* | 51.28732 | -2.75596 | Cheddar gorge | CG |
| 7 | V.2014.003.159 | *S. porrigentiformis* | 51.22465 | -3.81605 | Fishermans car park | WM |
| 47 | V.2014.003.161 | *S. porrigentiformis* | 51.46611 | -2.63517 | Leigh woods | LW |
| 533 |  | *S. porrigentiformis* |  |  | Luckbarrow ENHS (Torbay) |  |
| 67 | V.2014.003.153 | *S. porrigentiformis* | 50.4742 | -3.50361 | Redgate, Torbay | TB |
| 188 |  | *S. porrigentiformis* | 50.47497 | -3.50271 | Redgate, Torbay | TB |
| 81 | V.2014.003.157 | *S. porrigentiformis* | 50.47382 | -3.50242 | Redgate, Torbay | TB |
| 82 | V.2014.003.155 | *S. porrigentiformis* | 50.47371 | -3.50248 | Redgate, Torbay | TB |
| 83 | V.2014.003.156 | *S. porrigentiformis* | 50.47336 | -3.50251 | Redgate, Torbay | TB |
| 84 | V.2014.003.183 | *S. porrigentiformis* | 50.47152 | -3.50211 | Redgate, Torbay | TB |
| 41 | V.2014.003.160 | *S. porrigentiformis* | 51.45721 | -2.63513 | Stokeleigh Camp | AG |
| 68 | V.2014.003.154 | *S. porrigentiformis* | 50.47691 | -3.50228 | Walls Hill, Torbay | TB |
| 14 |  | *S. porrigentiformis* | 51.22802 | -3.90778 | Woody Bay | WB |
| 910 |  | *S. porrigentiformis* | 51.78454 | -3.42401 | Darren Fach | PDF |
| B9-F10 |  | *S. rupicola* | 51.78454 | -3.42401 | Darren Fach | PDF |
| B9-910 |  | *S. rupicola* | 51.77683 | -3.42755 | Penmoelallt | PDF |
| 85 | V.2014.003.050 | *S. rupicola* | 50.47946 | -3.51364 | Babbacombe slopes, Torbay | TB |
| 87 | V.2014.003.055 | *S. rupicola* | 50.48036 | -3.51367 | Babbacombe slopes, Torbay | TB |
| 62 | V.2014.003.019 | *S. rupicola* | 50.40265 | -3.52442 | Churston, Torbay | TB |
| 63 | V.2014.003.144 | *S. rupicola* | 50.4013 | -3.52397 | Churston, Torbay | TB |
| 64 | V.2014.003.149 | *S. rupicola* | 50.40164 | -3.52092 | Churston, Torbay | TB |
| 65 | V.2014.003.051 | *S. rupicola* | 50.40312 | -3.52474 | Churston, Torbay | TB |
| 66 | V.2014.003.147 | *S. rupicola* | 50.40228 | -3.52467 | Churston, Torbay | TB |
| 172 | V.2014.003.052 | *S. rupicola* | 50.40248 | -3.52423 | Churston, Torbay | TB |
| 173 | V.2014.003.053 | *S. rupicola* | 50.40349 | -3.52598 | Churston, Torbay | TB |
| 267 |  | *S. rupicola* | 57.03369 | -4.19312 | Creagh Dhubh, Scotland |  |
| 121 |  | *S. rupicola* |  |  | Luckbarrow (Neck Wood) |  |
| 22 | V.2014.003.145 | *S. rupicola* | 51.21917 | -3.95852 | Neck wood | NW |
| 265 |  | *S. subcuneata* | 51.2205 | -3.49021 | Greencliff, Minehead | GC |
| 266 |  | *S. subcuneata* | 51.2205 | -3.49021 | Greencliff, Minehead | GC |
| 33 | V.2014.003.022 | *S. subcuneata* | 51.21227 | -3.47574 | Greenleigh Wood, Minehead | GL |
| 34 | V.2014.003.122 | *S. subcuneata* | 51.21227 | -3.47574 | Greenleigh Wood, Minehead | GL |
| 23 | V.2014.003.123 | *S. subcuneata* | 51.21917 | -3.95874 | Neck wood | NW |
| 2 | V.2014.003.121 | *S. subcuneata* | 51.22534 | -3.79637 | Watersmeet | WM |
| 4 | V.2014.003.077 | *S. subcuneata* | 51.22642 | -3.80044 | Watersmeet | WM |
| 58 | V.2014.003.078 | *S. subcuneata* | 51.22539 | -3.80008 | Watersmeet | WM |
| 59 | V.2014.003.120 | *S. subcuneata* | 51.22575 | -3.80083 | Watersmeet | WM |
| 144 | V.2014.003.125 | *S. subcuneata* | 51.22343 | -3.79845 | Watersmeet | WM |
| 145 | V.2014.003.103 | *S. subcuneata* | 51.22436 | -3.79837 | Watersmeet | WM |
| 146 | V.2014.003.107 | *S. subcuneata* | 51.2242 | -3.79833 | Watersmeet | WM |
| 147 | V.2014.003.102 | *S. subcuneata* | 51.22439 | -3.79816 | Watersmeet | WM |
| 157 | V.2014.003.114 | *S. subcuneata* | 51.22379 | -3.79796 | Watersmeet | WM |
| 165 | V.2014.003.111 | *S. subcuneata* | 51.22469 | -3.79219 | Watersmeet | WM |
| 166 | V.2014.003.109 | *S. subcuneata* | 51.22556 | -3.80064 | Watersmeet | WM |
| 167 | V.2014.003.112 | *S. subcuneata* | 51.22566 | -3.80075 | Watersmeet | WM |
| 181 | V.2014.003.075 | *S. subcuneata* | 51.22468 | -3.79828 | Watersmeet | WM |
| 182 | V.2014.003.056 | *S. subcuneata* | 51.22459 | -3.79833 | Watersmeet | WM |
| 186 | V.2014.003.117 | *S. subcuneata* | 51.22644 | -3.80035 | Watersmeet | WM |
| 187 | V.2014.003.116 | *S. subcuneata* | 51.22533 | -3.79862 | Watersmeet | WM |
| 212 |  | *S. subcuneata* | 51.22394 | -3.79374 | Watersmeet | WM |
| 226 |  | *S. subcuneata* | 51.22453 | -3.79459 | Watersmeet | WM |
| 269 |  | *S. subcuneata* | 51.22365 | -3.79811 | Watersmeet | WM |
| 280 |  | *S. subcuneata* | 51.22493 | -3.79729 | Watersmeet | WM |
| 235 | V.2014.003.115 | *S. subcuneata* | 51.22144 | -3.8989 | Woody Bay | WB |
| 237 | V.2014.003.113 | *S. subcuneata* | 51.22839 | -3.90739 | Woody Bay | WB |
| 106 | V.2014.003.037 | *S. torminalis* | 50.91148 | -4.0721 | Beaford |  |
| 251 |  | *S. torminalis* | 50.716897 | -4.408505 | Beardon |  |
| 261 |  | *S. torminalis* | 50.707611 | -3.716723 | Berryhead plantation |  |
| 273 |  | *S. torminalis* | 50.52844 | -3.63954 | Broadridge wood, Newton Abbot | NA |
| 256 |  | *S. torminalis* | 50.876113 | -4.5402991 | Coombe Valley, nr stibb |  |
| 250 |  | *S. torminalis* | 50.503475 | -4.2524213 | Halton Barton |  |
| 243 | V.2014.003.046 | *S. torminalis* | 50.83352 | -4.10751 | Hatherleigh |  |
| 242 |  | *S. torminalis* | 51.64024 | -4.1388192 | Hatherleigh |  |
| 245 |  | *S. torminalis* | 50.830912 | -4.1418027 | Highampton |  |
| 247 |  | *S. torminalis* | 50.832512 | -4.1349456 | Highampton |  |
| 255 |  | *S. torminalis* | 50.869228 | -4.5317944 | Houndapitt, Nr Stibb |  |
| 42 | V.2014.003.135 | *S. torminalis* | 51.46256 | -2.63963 | Leigh woods | LW |
| 232 |  | *S. torminalis* | 50.562964 | -3.533977 | Little Haldon | LH |
| 241 |  | *S. torminalis* | 50.787998 | -3.817363 | Little Langford |  |
| 95 |  | *S. torminalis* |  |  | Luckbarrow ENHS collection |  |
| 94 |  | *S. torminalis* | 51.203371 | -3.5844902 | Luckbarrow, ENHS |  |
| 252 | V.2014.003.048 | *S. torminalis* | 50.76732 | -4.48058 | Odd Mill |  |
| 254 | V.2014.003.049 | *S. torminalis* | 50.76711 | -4.47862 | Odd Mill |  |
| 249 | V.2014.003.127 | *S. torminalis* | 50.44776 | -4.30243 | Pillaton Mill |  |
| 260 |  | *S. torminalis* | 50.666518 | -3.7051913 | Plaston Green |  |
| 69 | V.2014.003.039 | *S. torminalis* (3n) | 50.7294 | -3.9037 | South Tawton |  |
| 74 | V.2014.003.042 | *S. torminalis* | 50.7547 | -3.83881 | Spreyton |  |
| 75 |  | *S. torminalis* | 50.758138 | -3.8243868 | Spreyton |  |
| 76 |  | *S. torminalis* | 50.761542 | -3.8136719 | Spreyton |  |
| 77 |  | *S. torminalis* | 50.769495 | -3.8169577 | Spreyton |  |
| 259 |  | *S. torminalis* | 50.764303 | -3.8400693 | Spreyton |  |
| 49 |  | *S. torminalis* | 51.841978 | -2.6377618 | Symonds Yat | SY |
| 257 | V.2014.003.138 | *S. torminalis* | 50.85228 | -4.52043 | Tiscott- nr Stibb |  |
| 117 |  | *S. torminalis* | 51.008554 | -4.2065583 | Upcott |  |
| 116 |  | *S. torminalis* | 51.008409 | -4.2066227 | Upcott |  |
| 114 | V.2014.003.034 | *S. torminalis* | 51.00523 | -4.20671 | Upcott Wood |  |
| 115 | V.2014.003.036 | *S. torminalis* | 51.0052 | -4.20681 | Upcott Wood |  |
| 96 |  | *S. vexans* | 51.22402 | -3.66306 | Culbone | CB |
| 97 |  | *S. vexans* | 51.22459 | -3.66623 | Culbone | CB |
| 98 |  | *S. vexans* | 51.22471 | -3.66642 | Culbone | CB |
| 99 |  | *S. vexans* | 51.2247 | -3.6665 | Culbone | CB |
| 100 |  | *S. vexans* | 51.22471 | -3.66642 | Culbone | CB |
| 199 |  | *S. vexans* | 51.22471 | -3.66685 | Culbone | CB |
| 200 |  | *S. vexans* | 51.22434 | -3.66681 | Culbone | CB |
| 201 |  | *S. vexans* | 51.22442 | -3.6667 | Culbone | CB |
| 202 |  | *S. vexans* | 51.22442 | -3.6667 | Culbone | CB |
| 205 |  | *S. vexans* | 51.22501 | -3.66645 | Culbone | CB |
| 32 | V.2014.003.148 | *S. vexans* | 51.23509 | -3.75604 | Desolation | DL |
| 28 | V.2014.003.057 | *S. vexans* | 51.23385 | -3.75227 | Dogsworthy Combe, nr Desolation | DL |
| 8 | V.2014.003.064 | *S. vexans* | 51.22456 | -3.81578 | Fishermans car park | WM |
| 20 | V.2014.003.063 | *S. vexans* | 51.21893 | -3.95847 | Neck wood | NW |
| 21 | V.2014.003.059 | *S. vexans* | 51.21893 | -3.95847 | Neck wood | NW |
| 60 | V.2014.003.001 | *S. vexans* | 51.22595 | -3.82097 | Oxen tor | OT |
| 61 | V.2014.003.025 | *S. vexans* | 51.226 | -3.82156 | Oxen tor | OT |
| 133 | V.2014.003.023 | *S. vexans* | 51.22615 | -3.82165 | Oxen tor | OT |
| 134 | V.2014.003.028 | *S. vexans* | 51.2291 | -3.83256 | Oxen tor | OT |
| 135 | V.2014.003.024 | *S. vexans* | 51.22613 | -3.82128 | Oxen tor | OT |
| 136 | V.2014.003.026 | *S. vexans* | 51.22609 | -3.82127 | Oxen tor | OT |
| 138 | V.2014.003.029 | *S. vexans* | 51.22611 | -3.82171 | Oxen tor | OT |
| 139 | V.2014.003.027 | *S. vexans* | 51.22611 | -3.82164 | Oxen tor | OT |
| 9 | V.2014.003.060 | *S. vexans* | 51.22148 | -3.89927 | Woody Bay | WB |

**Table S2**. Multiplex design for PCR reaction of *Sorbus.*

| **Multiplex** | **Marker** | **Final concentration in PCR (µM)** | **Dilution for capillary electrophoresis** |
| --- | --- | --- | --- |
| MPLX 1 | CH01F02 | 0.06 | 20% |
|  | SA01 | 1.25 |  |
|  | SA19.1 | 1.25 |  |
|  | MSS5 | 0.375 |  |
|  | MSS16 | 0.125 |  |
| MPLX 2 | CH01F09 | 0.375 | 0% |
|  | CH02D11 | 0.125 |  |
|  | SA03 | 0.25 |  |
|  | SA06 | 0.125 |  |
|  | MSS13 | 0.25 |  |
| MPLX 3 | SA02 | 0.125 | 0% |
|  | SA08 | 0.075 |  |
|  | SA09 | 0.125 |  |
|  | MS14 | 0.025 |  |
| SINGLE | SA14 | 0.25 | 80% |
| **PCR 10 sample reaction mix:** 50 µl HotStarTaq Master mix, 10 µl MPLX primer mix, 30µl water.  1 µl DNA sample template + 9 µl PCR reaction mix | | | **Capillary electrophoresis mix:** 25µl SLS + internal size standard, 5 µl PCR product at specified dilution. |

**Table S3.** Genome composition of SW endemic taxa at ten loci plus the two genome specific loci. *X* = ploidy level. Allele sizes are from the majority (80%) genotype for polyploid taxa.

|  |  |  | Microsatellite loci | | | | | | | | | | | | | | | |  | |  | |
| --- | --- | --- | --- | --- | --- | --- | --- | --- | --- | --- | --- | --- | --- | --- | --- | --- | --- | --- | --- | --- | --- | --- |
| Taxon | *X* | CH01F02 | | | | | MSS16 | | | | | SA01 | | | | | MSS5 | | | | | |
| *S. admonitor* | 4 | 187 | |  | 195 | 199 | 158 | 160 | 188 | 204 | 224 | | 232 |  | 242 | 119 | | 121 | | 123 | | 127 |
| *S. devoniensis* | 4 | 187 | |  | 195 | 199 | 158 | 160 | 198 | 204 | 224 | | 232 | 234 | 242 | 119 | | 121 | | 123 | | 127 |
| *S. subcuneata* | 3 | 187 | |  | 195 | 199 | 158 | 160 |  | 204 | 224 | | 232 |  | 242 | 119 | | 121 | | 123 | |  |
| *S. margaretae* | 4 | 191 | | 195 | 199 | 221 | 158 | 160 | 162 |  | 224 | | 232 |  |  | 119 | | 121 | |  | | 135 |
| *S. vexans* | 4 | 191 | | 195 | 201 | 203 | 158 | 160 | 162 |  | 236 | |  |  |  | 119 | | 121 | |  | | 127 |
| *S. vexans* (vex2) | 4 | 191 | | 195 | 203 | 221 | 158 | 160 |  |  | 232 | | 236 | 238 |  | 121 | | 127 | | 129 | | 135 |
| *S. porrigentiformis* | 4 | 191 | | 197 | 201 | 203 | 158 | 162 | 170 |  | 214 | | 236 | 244 |  | 115 | | 127 | | 131 | | 137 |
| *S. rupicola* | 4 | 191 | | 199 | 201 | 209 | 158 | 162 |  |  | 224 | | 230 | 234 | 236 | 119 | | 127 | | 131 | |  |
| *S. torminalis* | 2 | 157 | | 167 | 175 | 187 | 154 | 166 | 170 | 178 | 190 | | 192 | 212 | 216 | 105 | | 113 | | 117 | | 119 |
|  |  | 189 | | 209 | 191 | 195 | 182 | 184 | 186 | 188 | 226 | | 230 | 234 | 236 | 123 | | 125 | | 127 | | 129 |
|  |  |  | |  |  |  | 190 | 194 | 196 | 198 | 238 | | 240 | 242 | 244 | 135 | | 137 | | 139 | | 141 |
|  |  |  | |  |  |  | 200 | 202 | 204 | 206 | 246 | | 256 |  |  |  | |  | |  | |  |
|  |  |  | |  |  |  | 208 | 210 | 216 | 222 |  | |  |  |  |  | |  | |  | |  |
|  |  |  | |  |  |  | 230 | 192 | 214 |  |  | |  |  |  |  | |  | |  | |  |
| *S. aria* | 2 | 191 | | 195 | 197 | 201 | 156 | 158 | 160 | 164 | 212 | | 220 | 230 | 232 | 115 | | 121 | | 127 | | 129 |
|  |  | 193 | | 207 |  |  |  |  |  |  | 234 | | 240 | 242 | 246 | 135 | | 139 | | 141 | |  |

|  |  | | Microsatellite loci | | | | | | | | | | | | | | | | | |
| --- | --- | --- | --- | --- | --- | --- | --- | --- | --- | --- | --- | --- | --- | --- | --- | --- | --- | --- | --- | --- |
| Taxon | | *X* | CH02D11 | | | | SA03 | | | | | SA06 | | | | | MSS13 | | | |
| *S. admonitor* | | 4 | 152 | 162 | 182 |  | 224 |  |  |  | 258 | | 268 |  |  | 187 | | 193 | 195 |  |
| *S. devoniensis* | | 4 | 152 | 162 | 182 |  | 224 |  |  |  | 258 | | 268 |  |  | 189 | | 193 | 195 |  |
| *S. subcuneata* | | 3 | 152 | 162 | 182 |  | 224 |  |  |  | 258 | | 268 |  |  | 193 | | 195 |  |  |
| *S. margaretae* | | 4 | 152 | 154 | 182 |  | 224 |  |  |  | 258 | | 268 | 280 | 312 | 193 | | 195 | 197 |  |
| *S. vexans* | | 4 | 150 | 154 |  |  | 224 | 240 |  |  | 258 | | 280 | 302 | 312 | 193 | | 195 | 197 | 199 |
|  | |  |  |  |  |  |  |  |  |  | 294 | | 282 |  |  |  | |  |  |  |
| *S. vexans* (vex2) | | 4 | 152 | 154 | 182 |  | 224 |  |  |  | 258 | | 264 | 268 | 278 | 193 | | 195 | 199 |  |
| *S. porrigentiformis* | | 4 | 152 | 198 |  |  | 228 | 240 |  |  | 256 | | 264 | 270 |  | 193 | | 195 | 197 | 203 |
| *S. rupicola* | | 4 | 152 | 162 | 168 |  | 224 | 240 |  |  | 258 | | 264 | 280 | 282 | 193 | | 195 | 197 |  |
|  | |  |  |  |  |  |  |  |  |  | 300 | | 312 | 302 |  |  | |  |  |  |
| *S. torminalis* | | 2 | 148 | 150 | 152 | 154 | 214 | 224 | 234 |  | 258 | | 260 | 268 | 270 | 181 | | 183 | 187 | 189 |
|  | |  | 156 | 162 | 164 | 170 |  |  |  |  | 278 | | 308 | 310 |  | 191 | | 193 | 195 | 197 |
|  | |  | 172 | 176 | 178 | 194 |  |  |  |  |  | |  |  |  |  | |  |  |  |
|  | |  | 196 | 174 | 193 | 200 |  |  |  |  |  | |  |  |  |  | |  |  |  |
| *S. aria* | | 2 | 154 | 156 | 164 | 172 | 224 | 240 | 242 | 250 | 256 | | 258 | 260 | 268 | 189 | | 195 | 197 | 199 |
|  | |  | 186 | 150 | 174 | 176 | 252 | 253 | 254 |  | 280 | | 282 | 288 |  | 203 | |  |  |  |
|  | |  | 180 |  |  |  |  |  |  |  |  | |  |  |  |  | |  |  |  |

|  |  | Microsatellite loci | | | | | | | | | | | | | | | |
| --- | --- | --- | --- | --- | --- | --- | --- | --- | --- | --- | --- | --- | --- | --- | --- | --- | --- |
| Taxon | *X* | SA14 | | | | SA08 | | | | SA09 | | | | SA02 | | | |
| *S. admonitor* | 4 | 170 | 178 | 208 | 226 | 261 | 285 |  |  | 162 | 194 |  |  | 292 | 294 |  |  |
| *S. devoniensis* | 4 | 170 | 204 | 208 | 226 | 261 |  |  |  | 162 | 194 |  |  | 292 | 294 |  |  |
| *S. subcuneata* | 3 | 170 |  | 208 | 226 | 261 |  |  |  | 162 | 194 |  |  | 292 | 294 |  |  |
| *S. margaretae* | 4 | 194 |  | 208 | 226 | 247 | 263 |  |  | 162 | 182 | 194 |  | 278 | 282 | 292 | 294 |
| *S. vexans* | 4 |  | 206 | 224 | 226 | 247 | 263 | 275 |  | 162 | 176 | 178 | 194 | 286 | 292 | 294 |  |
| *S. vexans* (vex2) | 4 | 204 | 212 | 224 | 226 | 247 | 275 |  |  | 160 | 178 | 182 | 194 | 278 | 286 | 294 | 300 |
| *S. porrigentiformis* | 4 | 196 | 222 | 224 | 226 | 249 | 257 | 277 |  | 174 | 176 | 184 | 186 | 294 | 300 | 324 |  |
| *S. rupicola* | 4 | 194 | 206 | 208 |  | 263 | 277 |  |  | 162 | 164 | 176 |  | 282 | 292 |  |  |
| *S. torminalis* | 2 | 170 | 176 | 178 | 180 | 229 | 232 | 259 | 260 | 162 | (triploid specimen) | | | 268 | 322 |  |  |
|  |  | 182 | 184 | 186 | 188 | 261 | 265 | 267 | 269 |  |  |  |  |  |  |  |  |
|  |  | 190 | 198 | 200 | 202 | 271 | 273 | 277 | 281 |  |  |  |  |  |  |  |  |
|  |  | 204 | 206 | 208 | 210 | 283 | 285 |  |  |  |  |  |  |  |  |  |  |
|  |  | 212 | 214 | 224 | 226 |  |  |  |  |  |  |  |  |  |  |  |  |
| *S. aria* | 2 | 194 | 202 | 210 | 212 | 249 | 251 | 253 | 259 | 162 | 186 | 176 | 188 | 280 | 220 | 292 | 230 |
|  |  | 216 | 230 | 240 | 242 | 273 | 275 | 277 |  |  |  |  |  | 322 | 289 | 264 | 279 |
|  |  | 258 |  |  |  |  |  |  |  |  |  |  |  | 290 | 286 | 285 | 324 |

|  |  | Microsatellite loci | | | | | | | | | | | |
| --- | --- | --- | --- | --- | --- | --- | --- | --- | --- | --- | --- | --- | --- |
| Taxon | *X* | SA19.1 | | | | CH01F09 | | | | MS14 | | | |
| *S. admonitor* | 4 | 224 | 232 |  |  | 113 | 123 |  |  | 123 | 131 |  |  |
| *S. devoniensis* | 4 | 224 | 232 |  |  | 113 | 123 |  |  | 123 | 133 |  |  |
| *S. subcuneata* | 3 | 224 | 232 |  |  | 113 | 123 |  |  | 123 |  |  |  |
| *S. margaretae* | 4 | 216 | 224 | 232 | 250 | 113 | 115 | 123 |  |  |  |  |  |
| *S. vexans* | 4 | 216 | 224 | 236 | 250 | 115 |  | 121 |  |  |  |  |  |
| *S. vexans* (vex2) | 4 | 224 | 234 | 236 | 256 | 115 |  | 123 |  |  |  |  |  |
| *S. porrigentiformis* | 4 | 216 | 228 | 238 |  | 115 | 123 | 125 | 129 |  |  |  |  |
| *S. rupicola* | 4 | 216 | 250 |  |  | 113 | 115 | 121 |  |  |  |  |  |
| *S. torminalis* | 2 | 222 | 224 | 226 |  |  |  |  |  | 122 | 123 | 125 | 127 |
|  |  | (triploid specimen) | | | |  |  |  |  | 129 | 131 | 133 | 135 |
| *S. aria* | 2 | 226 | 236 | 230 | 234 | 111 | 115 | 117 | 121 |  |  |  |  |
|  |  |  |  |  |  | 125 | 133 |  |  |  |  |  |  |
|  |  |  |  |  |  |  |  |  |  |  |  |  |  |


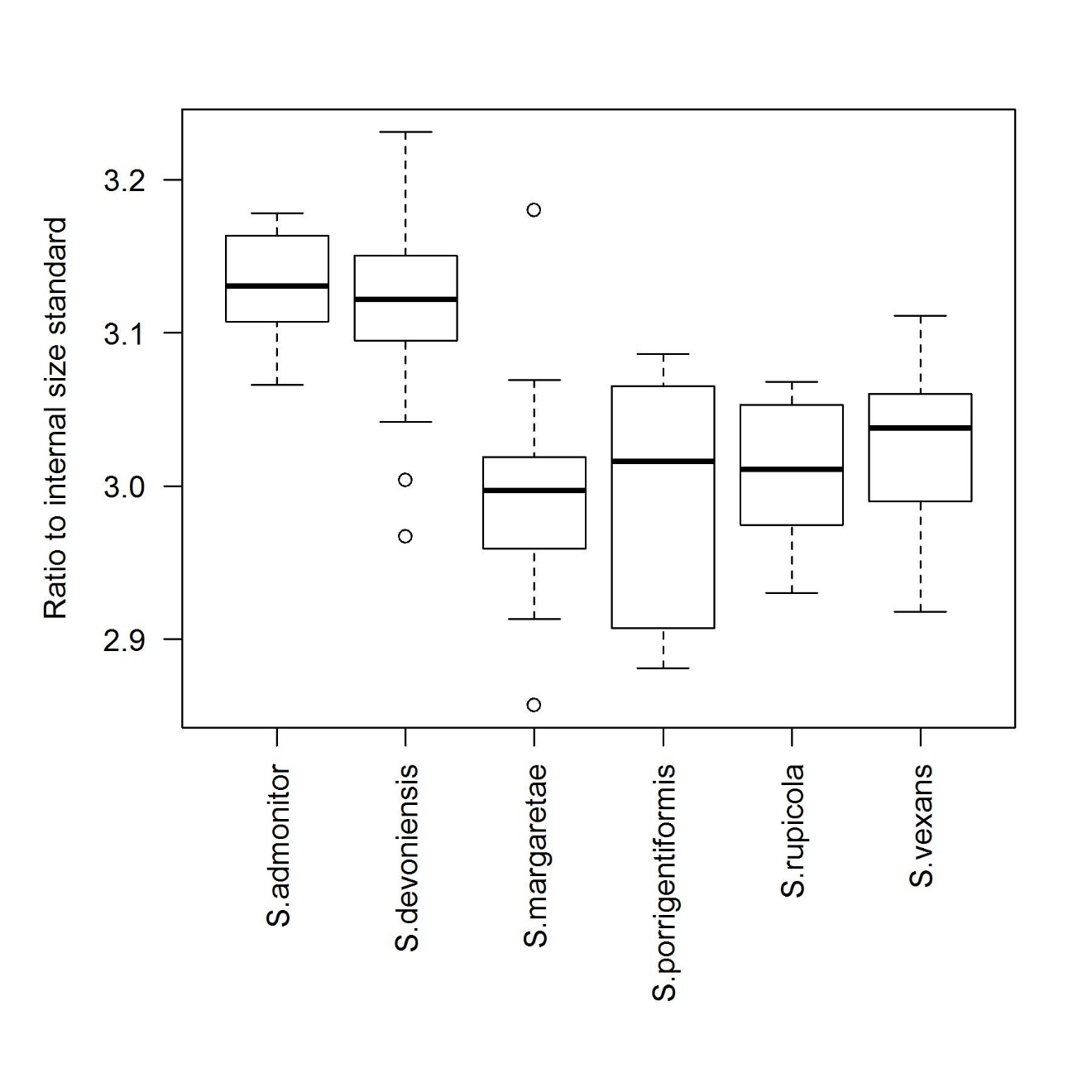


**Figure S1.** Relative nuclear DNA content of the tetraploid species. a and b indicate which comparisons were statistically significant in Tukey post hoc pairwise analysis with p< 0.01. Sample sizes = 12, 31, 13, 7, 11, 13, left to right.
